# Improving pentose fermentation by preventing ubiquitination of hexose transporters in *Saccharomyces cerevisiae*

**DOI:** 10.1186/s13068-016-0573-3

**Published:** 2016-07-26

**Authors:** Jeroen G. Nijland, Erwin Vos, Hyun Yong Shin, Paul P. de Waal, Paul Klaassen, Arnold J. M. Driessen

**Affiliations:** 1 Molecular Microbiology, Groningen Biomolecular Sciences and Biotechnology, University of Groningen, Zernike Institute for Advanced Materials and Kluyver Centre for Genomics of Industrial Fermentation, Groningen, The Netherlands; 2 DSM Biotechnology Center, Alexander Fleminglaan 1, 2613 AX Delft, The Netherlands

**Keywords:** Sugar transporter, Ubiquitination, Xylose transport, Yeast

## Abstract

**Background:**

Engineering of the yeast *Saccharomyces cerevisiae* for improved utilization of pentose sugars is vital for cost-efficient cellulosic bioethanol production. Although endogenous hexose transporters (Hxt) can be engineered into specific pentose transporters, they remain subjected to glucose-regulated protein degradation. Therefore, in the absence of glucose or when the glucose is exhausted from the medium, some Hxt proteins with high xylose transport capacity are rapidly degraded and removed from the cytoplasmic membrane. Thus, turnover of such Hxt proteins may lead to poor growth on solely xylose.

**Results:**

The low affinity hexose transporters Hxt1, Hxt36 (Hxt3 variant), and Hxt5 are subjected to catabolite degradation as evidenced by a loss of GFP fused hexose transporters from the membrane upon glucose depletion. Catabolite degradation occurs through ubiquitination, which is a major signaling pathway for turnover. Therefore, N-terminal lysine residues of the aforementioned Hxt proteins predicted to be the target of ubiquitination, were replaced for arginine residues. The mutagenesis resulted in improved membrane localization when cells were grown on solely xylose concomitantly with markedly stimulated growth on xylose. The mutagenesis also improved the late stages of sugar fermentation when cells are grown on both glucose and xylose.

**Conclusions:**

Substitution of N-terminal lysine residues in the endogenous hexose transporters Hxt1 and Hxt36 that are subjected to catabolite degradation results in improved retention at the cytoplasmic membrane in the absence of glucose and causes improved xylose fermentation upon the depletion of glucose and when cells are grown in d-xylose alone.

**Electronic supplementary material:**

The online version of this article (doi:10.1186/s13068-016-0573-3) contains supplementary material, which is available to authorized users.

## Background

During the last three decades, biofuels have received a lot of attention since these are derived from renewable lignocellulosic biomass and can potentially replace conventional fossil fuels. However, the lignocellulosic biomass (from hardwood, softwood, and agricultural residues) which is used to produce bioethanol contains up to 30 % of xylose next to the glucose [1]. In industrial fermentation processes, *Saccharomyces cerevisiae* is generally used for ethanol production but this yeast cannot naturally ferment pentose sugars, like, xylose. Although *S. cerevisiae* has been engineered into a xylose-fermenting strain via either the insertion of a xylose reductase and xylitol dehydrogenase [2, 3] or a fungal xylose isomerase [4–6], glucose remains the preferred sugar which is consumed first. Therefore, during growth of contemporary xylose-fermenting *S. cerevisiae* strains on a second generation feed stock, consumption rates of xylose in the presence of high glucose concentrations always remained moderate [7]. Instead, bi-phase sugar consumption is observed which relates to sequential sugar uptake wherein first glucose and subsequently xylose is sequestered by the cells. All wild-type *S. cerevisiae* hexose transporter (Hxt) proteins show a higher *K*_m_ value for xylose uptake as compared to glucose which explains the preference for glucose over xylose [7, 8]. More recently, co-fermentation of these sugars has been reported through the engineering of endogenous Hxt transporters (Hxt’s) [9, 10] yielding non-glucose inhibited xylose transporters. A Hxt-deletion strain, in which the *HXT1*-*17* and *GAL2* genes were removed, is unable to grow on xylose and glucose while growth on xylose could be complemented with Hxt4, Hxt5, Hxt7, and Gal2 [7]. Saloheimo and coworkers [8] additionally showed that also Hxt1 and Hxt2 are able to transport xylose in a strain in which the main hexose transporter genes *HXT1*-*7* and *GAL2* were deleted. In none of these studies, Hxt3 was analyzed. In general, the HXT family of sugar transporters facilitates glucose transport in *S. cerevisiae* [11, 12] while Hxt1-7 and Gal2 are the main and highest expressed transporters exhibiting different affinities for glucose thus covering a wide range of glucose concentrations [12, 13]. Hxt transporters can be divided into three groups on the basis of their glucose transporter affinity and expression, namely: low affinity glucose transporters Hxt1 and Hxt3 (*K*_m_ 40–100 mM) which are expressed at high glucose concentrations but disappear from the membrane at low glucose concentration; moderate affinity glucose transporters Hxt4 and Hxt5 (*K*_m_ 10–15 mM) with a varied expression profile; and high affinity transporters Hxt2 and Hxt7 (*K*_m_ 1–3 mM) that are solely expressed at lower glucose concentration [11, 13–16]. Gal2 which is a galactose transporter also shows a high affinity for glucose (*K*_m_ 1.5 mM) [9]. However, the *GAL2* gene is expressed only when galactose is present [13, 17]. Expression studies have shown that the *HXT1*-*4* genes are mainly repressed by the Rgt1 repressor, which recruits the general co-repressor Cyc8-Tup1 complex and the co-repressor Mth1 to the *HXT* promoters in the absence of glucose [18–23]. *HXT* genes are induced by three major glucose signaling pathways (Rgt2/Snf3, AMPK, and cAMP-PKA) which bring about glucose induction by inactivating the Rgt1 repressor [24–26] and as demonstrated for Hxt1 and Hxt3, subsequent endocytosis and vacuolar degradation of cytoplasmic membrane localized transporters when the glucose concentration is low [20, 27, 28]. Protein degradation in *S. cerevisiae* is brought about via the ubiquitination of the target proteins [28]. Ubiquitin is typically linked to the target protein through an isopeptide bond between the ɛ-amino group of a substrate lysine residue and the carboxyl terminus of ubiquitin [29]. Hxt1 has previously been shown to be ubiquitinated when the glucose levels in the medium are low [20].

A potential issue with the use of Hxt-derived, engineered xylose transporters is that their overexpression not always matches to the growth phase and/or carbon source under study. For example, if a xylose transporter would be derived from Hxt3 by protein engineering, one should note that Hxt3 intrinsically is a low-affinity glucose/xylose transporter induced at high glucose concentrations while the protein is rapidly degraded and removed from the plasma membrane in the absence of glucose [28]. Hxt3 indeed supports only limited or no growth when cells are supplied with solely xylose [7]. The Hxt1 [20] and Hxt3 [28] transporters have in common that upon depletion of glucose in the medium, they are removed from the membrane and for Hxt1 it was shown that it is indeed ubiquitinated at two lysine residues in the N-terminus [20]. This pathway involves endocytosis and vacuolar degradation. Hxt36 is a chimeric protein constituting the N-terminus of Hxt3 (438 amino acids) and the C-terminus of Hxt6 (130 amino acids). This chimeric protein occurs in specific xylose-consuming *S. cerevisiae* strains that have been evolved for industrial bioethanol formation [10]. Hxt36 is highly homologus to Hxt3, and, is like Hxt3 [28], also susceptible to degradation in the absence of glucose. Thus, the presence of the C-terminus of Hxt6 did not rescue Hxt36 against turnover. Moreover, in a Hxt-deficient strain, Hxt36 supported only slow xylose consumption in a fermentation in the absence of glucose [10]. Hxt5 is an intermediately expressed hexose transporter at low glucose concentration exhibiting a moderate affinity for glucose (~10 mM) [30] and is regulated by growth rate [14]. Hxt5 is degraded at high concentrations of glucose in the medium [31]. In stationary-phase cells, Hxt5 is transient phosphorylated on serine residues and no ubiquitination was detected [31]. As a possible strategy to prevent the protein degradation of the aforementioned transporters Hxt1, Hxt36, and Hxt5, we have mutated lysine residues predicted to be potential targets for ubiquitination and expressed these mutant proteins in xylose-fermenting yeast. The presented data show that mutagenesis results in a marked retention of these transporters at the cytoplasmic membrane both at high and low glucose concentration and improved growth on solely xylose in anaerobic fermentations. Thereby, a method is proposed to retain potentially interesting HXT-based, engineered transporters with affinity for xylose at the membrane in mixed sugar fermentations with varying glucose concentrations.

## Results

### Mutagenesis of putative ubiquitination sites on Hxt transporters and growth on xylose

**Fig. 1:**
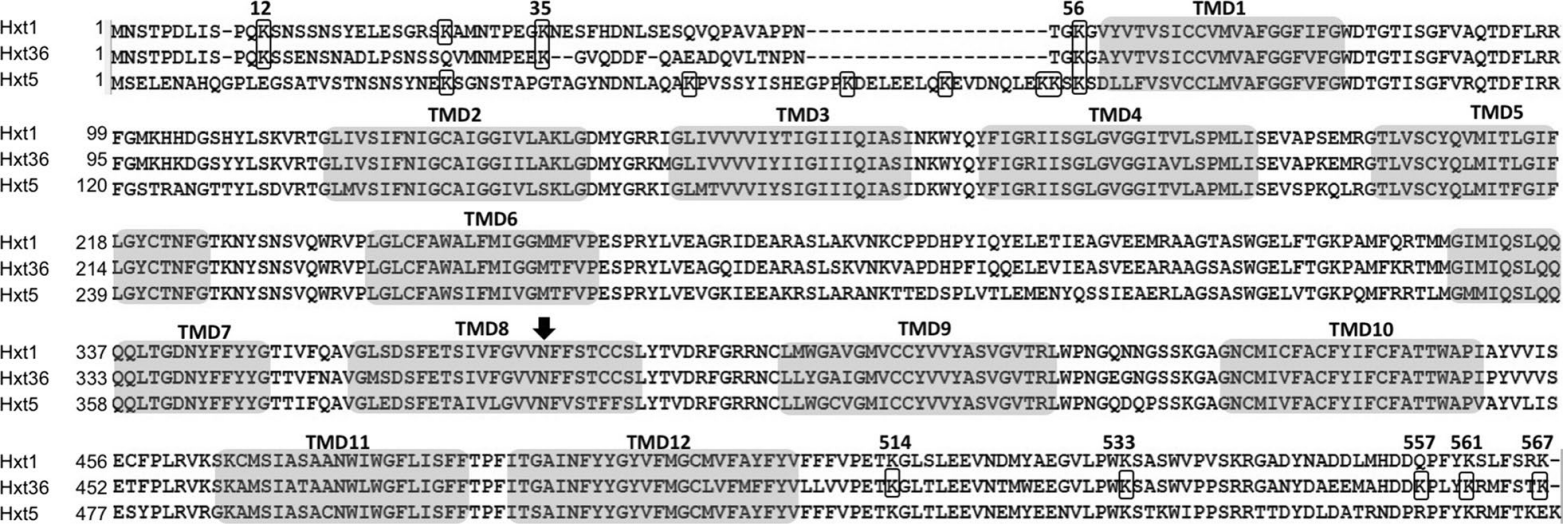
Alignment of Hxt1, Hxt36, and Hxt5 transporters. The lysine residues mutated in the respective hexose transporters are *boxed*, and the transmembrane domains (TMDs) are *shaded gray*. The position of the asparagine residue in Hxt36 that was mutated to an alanine to obtain Hxt36-N367A mutant is indicated with an *arrow*. Numbering of the targeted lysine residues is for Hxt36

**Fig. 2:**
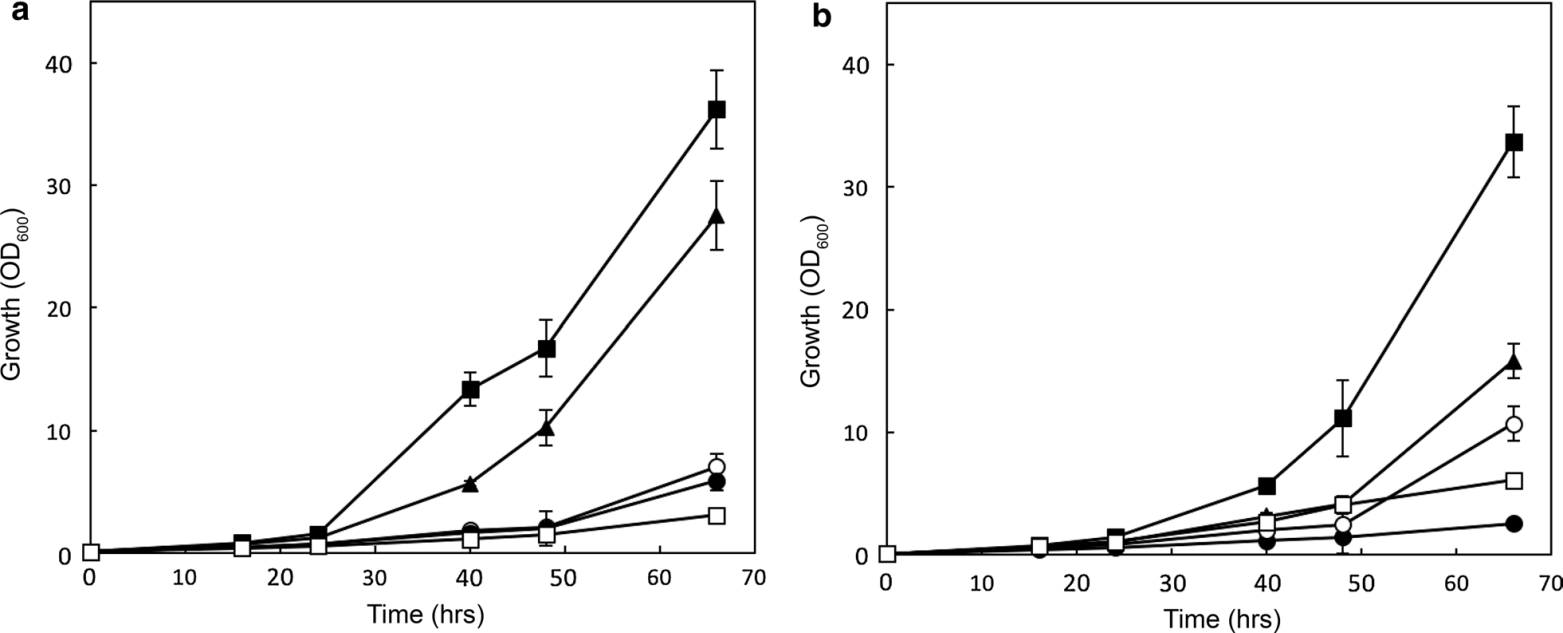
Growth of strain DS68625 over-expressing Hxt36 (**a**) and Hxt36 N367A (**b**) on 2 % d-xylose. Different variants of Hxt36 were used in which the N- and C-terminal lysine residues were replaced by arginine residues (*open square* wt, *open circle* 1K, *filled square* 3K, *filled circle* 5K, and *filled triangle* 8K)

Hxt transporters in *S. cerevisiae* are regulated both at the transcriptional and posttranslational level [16]. Here, individual HXT genes were removed from their native transcriptional regulation and constitutively expressed under control of the truncated (−390) *HXT7* promoter in a ∆*hxt1*-*7*; ∆*gal2* deletion strain with the aim to investigate the impact of the posttranslational degradation process on sugar fermentation. The Hxt36 amino acid sequence was analyzed for putative ubiquitination sites using UbPred (http://www.ubpred.org/) predicting possible ubiquitination of the lysine residues at position 12, 35, 420, 557, and 561 (Fig. 1; numbered according to Hxt1 that acts as a reference). Lysine 420 showed a low confidence score and is localized to an external loop of Hxt36. Therefore, we focused on the lysine positions in the N- and C-terminus of Hxt36. These residues were mutagenized to arginine, and various combinations of mutagenized Hxt36 proteins were generated. Via overlap-PCR the following combinations were made: K12R, K12,35,56R, K514,533,557,561,567R, and K12,35,56,514,533,557,561,567R that were cloned into pRS313-P7T7 [10] and renamed 1K, 3K 5K, and 8K, respectively. Furthermore, all aforementioned mutations were also introduced in the *HXT36* N367A gene, which enables co-fermentation of d-glucose and d-xylose due to an improved substrate specificity towards d-xylose over d-glucose [10]. All mutants and wild-type *HXT36* genes were transformed into strain DS68625 which lacks the *HXT1*-*7, GAL2* genes and is equipped with a xylose fermentation pathway [11]. Subsequently, cells were grown on 2 % d-xylose (Fig. 2). In this strain, growth on xylose is dependent on the introduction of a Hxt transporter. In the case of both Hxt36 variants, the triple lysine mutations in the N-terminus of Hxt36 (3K) enabled efficient growth on d-xylose as sole carbon source. Notably, with the mutant *HXT36* N367A gene, growth solely on d-xylose occurred with an increased lag-phase (Fig. 2b). The Hxt36 wild type bearing the mutations in all C- and N-terminal lysine residues (8K) also enabled improved growth on d-xylose but not as well as the 3K mutant (Fig. 2a). The 5K with mutations only in the C-terminus and the 1K mutant showed only small improvements. The data show that replacement of, in particular, the three N-terminal lysine residues of Hxt36 for arginine results in improved growth on d-xylose.

A similar approach was followed for Hxt1 and Hxt5, and the N-terminal lysine residues were replaced by arginine residues at positions 12, 27, 35, and 59; and 28, 48, 61, 69, 77, 78, and 80, respectively (Fig. 1). These mutants are further referred to as Hxt1 K4 and Hxt5 K7. All mutant proteins were cloned into pRS313-P7T7 and transformed to the DS68625 strain. In contrast to the wild-type Hxt1, the strain carrying the Hxt1 K4 mutant was capable of growth on 2 % d-xylose (Additional file 1: Figure S1A). The Hxt5 K7 mutant did not improve upon the wild-type Hxt5 (Additional file 1: Figure S1B) confirming an earlier study [31]. These data show that mutating potential ubiquitination sites on Hxt transporters unmasks the ability of such transporters to support growth of yeast on solely xylose. In the remainder of this study, we, therefore, focused on the Hxt36 3K mutant which showed the most prominent effect.

### Membrane localization and retention of mutated Hxt transporters

**Fig. 3:**
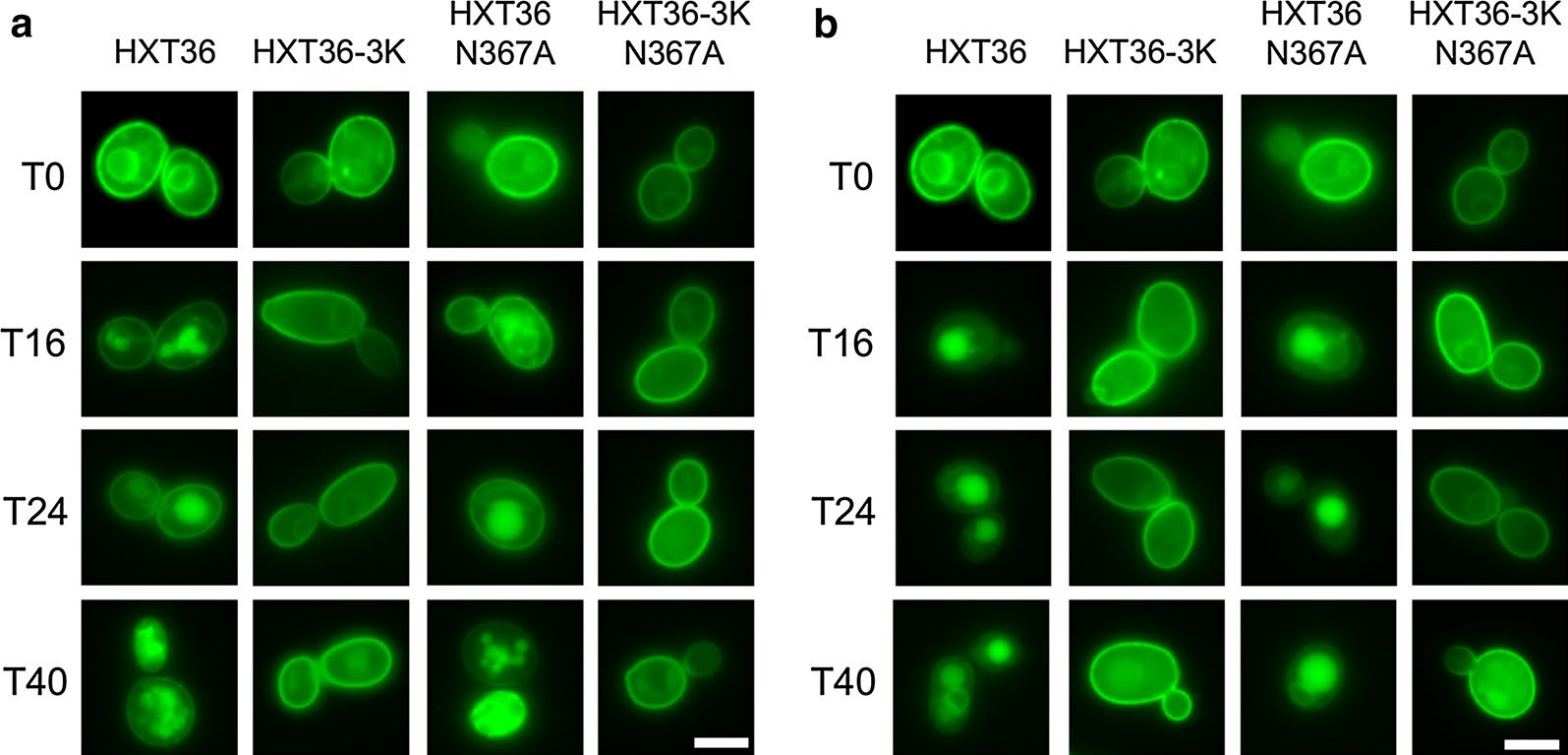
Membrane localization of Hxt36 and Hxt36 N367A fused to GFP with and without the 3K (K12,25,56R) mutations when grown on minimal medium with 2 % d-glucose (**a**) and 2 % d-xylose (**b**) in a 0–40 h time range. The *scale bar* corresponds to 5 μm

To determine if the improved growth on d-xylose relates to an improved retention of the mutated Hxt transporters at the cytoplasmic membrane, which is expected when turnover is prevented, the different mutants were fused C-terminally to the fluorescent reporter GFP. The various fusion proteins were expressed in the DS68625 strain, and cells were pre-grown with d-glucose and transferred to a medium with 2 % d-glucose or d-xylose. Next, at various time intervals, the cellular localization of the Hxt-GFP fusion protein was assessed by fluorescence microscopy. Since, the growth rate on d-xylose (Fig. 2) and on low concentrations of d-glucose (data not shown) by the respective lysine mutants was significantly increased as compared to the wild-type Hxt36 and Hxt36 N367A, it is difficult to obtain *S. cerevisiae* cells which are in exactly the same, active budding, state. Therefore, microscopic investigations were performed over a large time span of growth to observe the general trend. On d-glucose, Hxt36 was readily degraded as a major share of the GFP fluorescence was recovered in the vacuole already after 16 h of growth. At that time point, the d-glucose concentration was close to zero (data not shown). Progressively less GFP signal was retained on the plasma membrane over time and after 40 h hardly any GFP fluorescence could be localized at the cytoplasmic membrane (Fig. 3a). Wild-type Hxt36 supported only slow growth on xylose (Fig. 2). At the start of the growth experiment on d-xylose (Fig. 3b, T0), still, a plasma membrane signal was observed due to pre-culture conditions on glucose but later degradation was severe as after 16 h hardly any GFP could be localized to the cytoplasmic membrane (Fig. 3b). In contrast, the Hxt36-3K GFP fusion localized almost exclusively to the plasma membrane independent of carbon source and the time the strain was grown (Fig. 3a, b). The Hxt36 N367A mutant showed a similar phenotype as the wild-type protein, and thus mutagenesis of the N-terminal lysine residues also resulted in stable cytoplasmic membrane localization. Thus, the mutagenesis of the three lysines in this mutant had a marked effect on membrane retention of Hxt36. Wild-type Hxt1 was readily degraded when cells were grown on d-xylose or when the d-glucose was exhausted from the medium, whereas the Hxt1 K4 mutant stably localized to the cytoplasmic membrane, even after 40 h (Additional file 1: Figure S2A). In contrast, with the Hxt5-GFP fusion a slower protein degradation rate was noted when the d-glucose was utilized, and even after 40 h, still some of the GFP localized to the cytoplasmic membrane. Similar results were obtained when cells were grown on d-xylose (Additional file 1: Figure S2A). The Hxt5 K7 mutant shows improved retention (Additional file 1: Figure S2B) but this effect was not as marked as with Hxt36 and Hxt1. In line with this observation that Hxt5 is more stably expressed than Hxt36 and Hxt1 when cells are grown only on d-xylose, growth on d-xylose was not markedly improved by the Hxt5 K7 mutant (Additional file 1: Figure S2B). It is concluded that mutagenesis of the N-terminal lysine residues to arginine in Hxt36 and Hxt1 which is predicted to interfere with ubiquitination results in improved cytoplasmic membrane localization of the aforementioned transporters.

### Sugar uptake by the mutant Hxt proteins

**Fig. 4:**
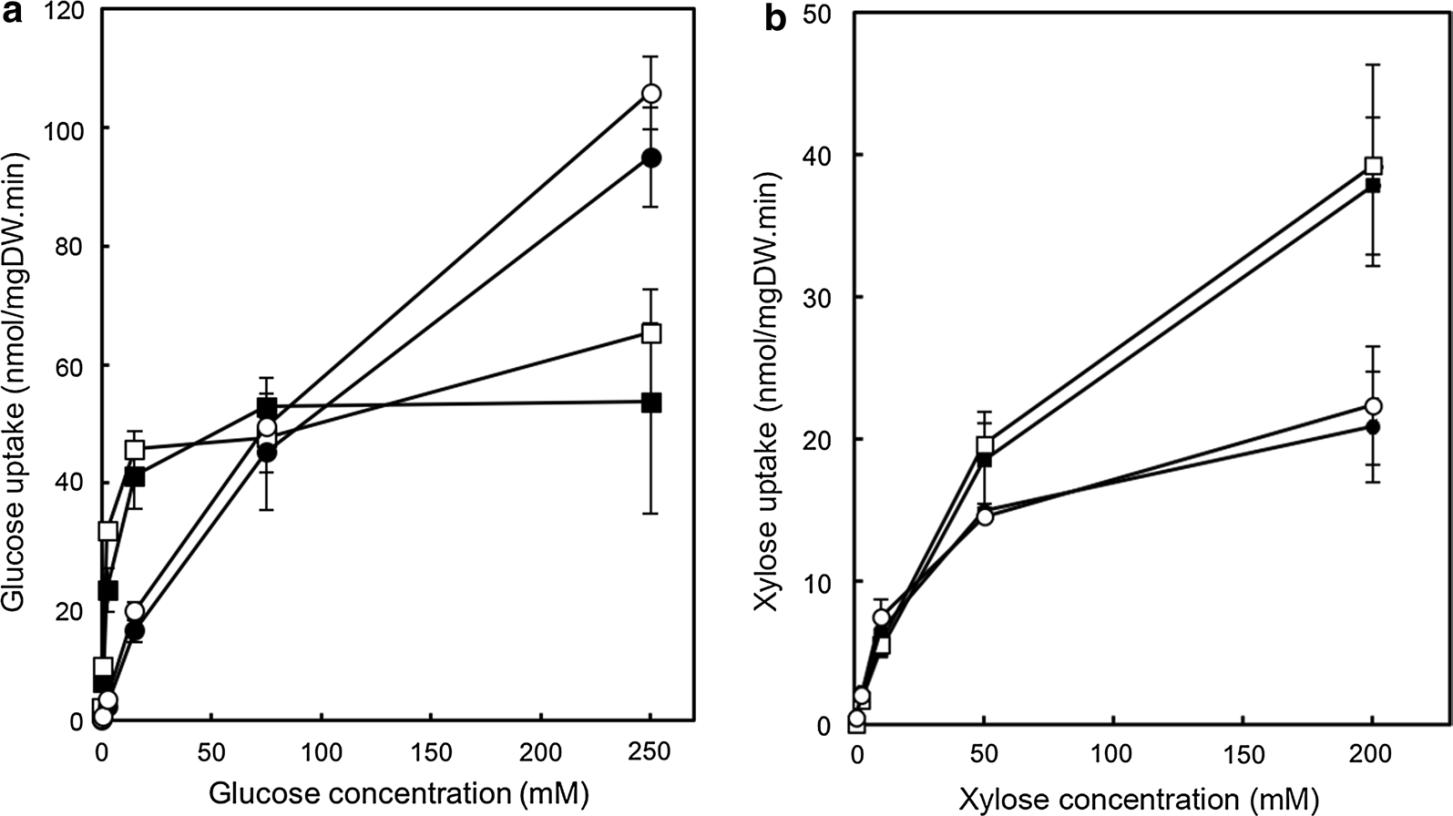
Kinetic analysis of the uptake of [^14^C-] d-glucose (**a**) and [^14^C-] d-xylose (**b**) by the DS68625 strain expressing Hxt36 (*filled square*), Hxt36-3K (*open square*), Hxt36 N367A (*filled circle*), and Hxt36 N367A-3K (*open circle*). *Errors* are the standard deviation of two independent experiments

The improved xylose fermentation characteristics of the cells bearing the Hxt36 and Hxt1 transporters with mutated ubiquitination sites is likely due to decreased protein degradation and hence more stable membrane localization. However, the mutations may also have altered the transport characteristics of the transporter. To circumvent the observed differences in protein degradation in the absence of d-glucose, and to assure identical growth conditions, all strains carrying the wild-type and mutated Hxt transporters were inoculated and grown in minimal medium containing 4 % d-maltose and grown for only 15 h to prevent depletion of the d-maltose. Although there is a marked difference in the uptake of d-glucose (Fig. 4a) and d-xylose (Fig. 4b) between the Hxt36 and the Hxt36 N367A mutant due to the altered substrate specificity [10], the N-terminal lysine mutations had little impact on the affinity of transport (Table 1). Compared to the previously described Hxt11 transporter [32], the *K*_m_ value for d-xylose by Hxt36 is similar (i.e., 71.8 ± 3.6 mM for Hxt36 vs 84.2 ± 10.0 mM for Hxt11) whereas the *V*_max_ for xylose transport is higher for Hxt11 (i.e., 48.1 ± 8.0 nmol/mgDW.min for Hxt36 vs 84.6 ± 2.4 nmol/mgDW.min for Hxt11) (Table 1). A similar difference in *V*_max_ is apparent when the mutated versions of Hxt36 (N367) and Hxt11 (N366) are compared (Table 1).

**Table 1:** *K*_m_ and *V*_max_ values for d-glucose and d-xylose uptake by various Hxt36 transporters expressed in the Hxt deletion strain DS68625 grown on maltose

	*K*_m_ (mM)		*V*_max_ (nmol/mg DW.min)	
	Glucose	Xylose	Glucose	Xylose
Hxt36	4.7 ± 2.5	71.8 ± 3.6	56.4 ± 11.5	48.1 ± 8.0
Hxt36-3K	2.6 ± 0.2	58.6 ± 1.3	58.0 ± 2.4	42.4 ± 1.1
Hxt36 N367A	165.7 ± 28.6	32.8 ± 11.5	116.4 ± 14.4	25.4 ± 5.1
Hxt36 N367A-3K	197.7 ± 14.6	34.2 ± 14.3	124.3 ± 10.2	26.7 ± 4.3
Hxt1^a^ Hxt11	33.4 ± 2.1	84.2 ± 10.0	156.4 ± 7.6	84.6 ± 2.4
N366T^a^	194.4 ± 47.9	46.7 ± 2.7	238.6 ± 7.4	76.2 ± 4.8

Errors are the standard deviation of two independent experiments

^a^From Ref. [34]

Hxt1 and Htx5 were not analyzed in detail with respect to the sugar transport kinetics, but uptake was assessed only at 100 mM of d-glucose or d-xylose, again using cells grown on maltose. Also in this case, the N-terminal lysine mutations did not affect the uptake (see Additional file 1: Figure S3A, B). These data show that the lysine mutations introduced in the N-terminus have little effect on the actual transporter affinity. Rather, the mutations affect stability of Hxt1 and Hxt36 in the absence of glucose, and thus will support increased transport rates on solely d-xylose.

### Sugar fermentations

**Fig. 5:**
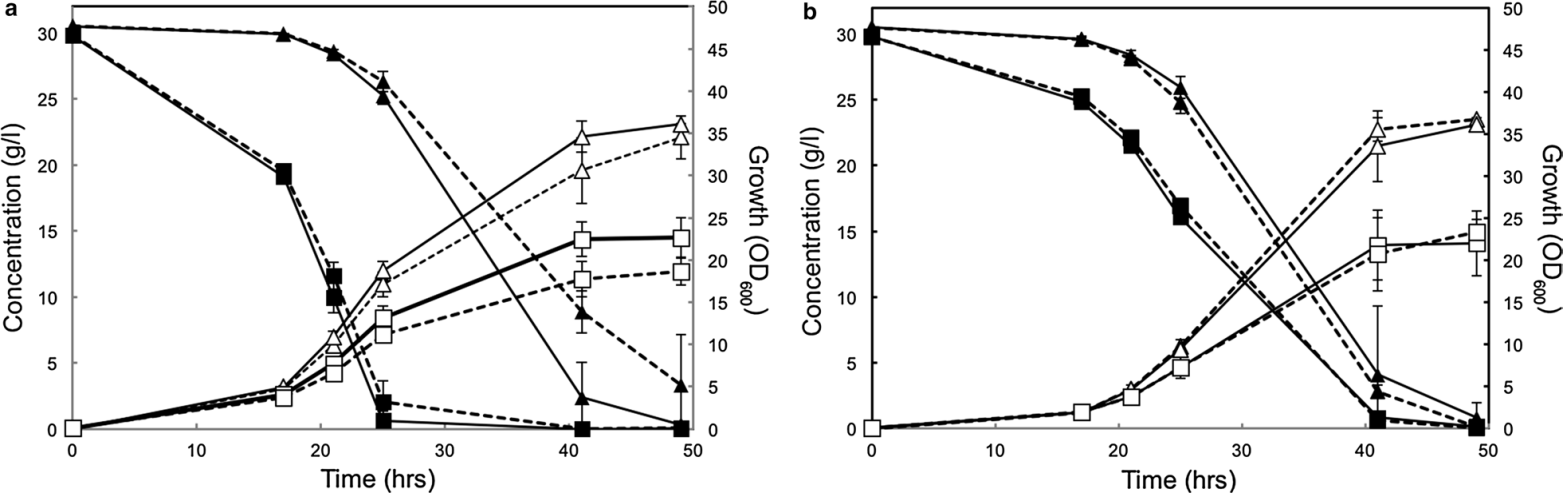
Fermentation of d-glucose and d-xylose by strain DS68625 expressing Hxt36 or Hxt36-3K (**a**) and Hxt36 N367A and Hxt36 N367A-3K (**b**). Symbols depicted show growth (OD_600_; *open squares*), ethanol (*open triangles*), d-glucose (*closed squares*), and d-xylose (*closed triangles*). The 3K mutants are shown as *solid lines*, and the parental transporters are indicated with *dashed lines*. *Errors* are the standard deviation of two independent experiments

To investigate if the low levels of Hxt protein degradation also impact the profile in mixed sugar fermentation, the Hxt36 wild-type and N367A mutant with and without the N-terminal lysine mutations were grown anaerobically with 3 % d-glucose and 3 % d-xylose. Wild-type Hxt36 supported the characteristic sequential consumption of d-glucose and d-xylose (Fig. 5a). At the end of fermentation, i.e., after 48 h, the d-xylose was not yet completely consumed (3.32 g/l d-xylose left). The Hxt36-3K mutant also showed bi-phase sugar consumption, but with improved d-xylose consumption such that at the end of the fermentation nearly all d-xylose were utilized with the concomitant increase in growth (Fig. 5a) and ethanol productivity (0.54 ± 0.03 g/l.h in Hxt36-3K vs 0.48 ± 0.04 g/l.h in Hxt36) (Additional file 1: Table S2). Compared to an earlier study using the wild-type and the N366A mutant of Hxt 11 [32], the ethanol productivity appears about three times lower in this study. However, the ethanol productivity is depending on the inoculation OD_600_ and the total sugar concentration, both of which were higher in the aforementioned study. Ethanol yield (Y_EtOH_) and specific ethanol production rate (Q _EtOH_) were, however, similar (Additional file 1: Table S2).

In contrast to Hxt36, the Hxt36 N367A variant and its 3K mutant, both, showed co-consumption of d-glucose and d-xylose, and there was no increased d-xylose consumption rate at the end of the fermentation (Fig. 5b). Both strains consumed all d-glucose and d-xylose within 48 h showing in the end, similar growth (Fig. 5a) and ethanol productivity as the Hxt36-3K mutant (Additional file 1: Table S2). It should be stressed that the greatest benefit by the lysine mutations in d-xylose consumption is expected in the strains that show bi-phasic sugar utilization, as d-xylose consumption commences only once the d-glucose is exhausted which in turn induces turnover of the respective transporters. When cells co-consume both sugars, d-xylose transport will hardly be affected as the presence of d-glucose will ensure membrane retention of the Hxt36 transporter. When grown solely on 3 % d-xylose, the ethanol production rate (Q _EtOH_) and ethanol productivity by both the 3K mutants were significantly increased compared to the Hxt36 wild-type and N367A mutant transporters (Additional file 1: Table S2). This is because the wild-type Hxt36 only supports poor growth on solely d-xylose (Fig. 2). The Q _EtOH_ is, however, corrected for the biomass and therefore less improved. On 3 % d-glucose, there are no major differences observed among the various transporters as expected as the transporters then remain at the membrane.

The yeast strains bearing Hxt1 and Hxt5 and their respective lysine mutants were also subjected to mixed sugar fermentation. Compared to Hxt36, Hxt1 showed an extended fermentation time but the Hxt1K4 mutant consumed the d-xylose faster compared to the wild type (Additional file 1: Figure S4A) also resulting in more biomass (OD_600_). The fermentation profile of Hxt5 is similar to Hxt36, but both Hxt5 and the Hxt5 K7 mutant consumed both sugars within 48 h. However, the remaining d-xylose was consumed somewhat faster by the Hxt5 K7 mutant (Additional file 1: Figure S4B) compared to the wild type in which the d-xylose consumption rates in the absence of d-glucose (at time points 24–32 h) were 1.35 g/h ± 0.16 and 1.18 ± 0.15 g/h, respectively. Taken together, these data suggest that mutations that prevents ubiquitination in Hxt36, Hxt1, and Hxt5 result in enhanced rates of xylose consumption in the late stages of fermentation when cells are grown on a mixture of d-xylose and d-glucose.

## Discussion

In the yeast *Saccharomyces cerevisiae,* the expressed transporters, Hxt1-7, function as facilitators for d-glucose. With a reduced affinity, these systems also mediate influx of d-xylose which is a critical step when cells grow on processed lignocellulosic biomass that contains both d-glucose and d-xylose. For industrial bioethanol production, xylose-fermenting *S. cerevisiae* strains are used but in such strains d-xylose consumption commences only when the d-glucose is exhausted. For a more robust fermentation, co-consumption of both sugars is desired. Although several *S. cerevisiae* strains metabolize d-xylose efficiently, uptake and, therefore, consumption of d-xylose is strongly inhibited by d-glucose [10]. Although, recently, some co-consumption could be observed depending on the specific *S. cerevisiae* strain examined [33], this problem is much more efficiently tackled by mutagenesis of endogenous Hxt transporters resulting in the specific d-xylose uptake even in the presence of d-glucose [9, 10, 32, 34]. However, many of the Hxt proteins are rapidly degraded in the absence of d-glucose [28] and therefore their capacity to mediate d-xylose transport is underestimated as these transporters are readily removed from the cytoplasmic membrane by a mechanism that involves the ubiquitination-dependent degradation pathway once the d-glucose is depleted in the medium. Here, we have shown that catabolite degradation of the chimeric Hxt36 transporter can be overcome by substituting the three N-terminally located lysine residues (3K) for arginine which should effectively prevent ubiquitination. This mutagenesis indeed has a major effect on the growth of *S. cerevisiae* on solely d-xylose. To sustain growth on d-xylose, small amounts of d-maltose were needed [10] to shorten the lag phase of cells bearing Hxt36 grown on 2 % d-xylose. Although this allowed a more rapid start of growth, overall growth on d-xylose remained limited. In contrast, the Hxt36-3K mutant used in the current study supports rapid growth even without the addition of maltose. In an industrial-like fermentation, with high levels of d-glucose and d-xylose, the Hxt36-3K mutant showed sequential utilization of d-glucose and d-xylose, but the overall fermentation time was slightly shortened due to an increased rate of d-xylose in the absence of d-glucose at the end of the fermentation. In contrast, our previously reported Hxt36 N367A mutant [10] expressed in the DS68625 strain shows co-consumption of d-glucose and d-xylose. With this mutant, the lysine mutations did not affect the overall fermentation likely because d-glucose remained present throughout the fermentation. Under those conditions, Hxt36 will remain on the membrane and thus sustain co-consumption. Using GFP fusions to detect the membrane localization and expression of Hxt36, the mutagenesis of the N-terminal lysine residues was indeed found to stabilize the expression of Hxt36 and Hxt36 N367A at the cytoplasmic membrane when cells are grown only on d-xylose which is consistent with the presumed role of ubiquitination in catabolite degradation.

A recent study that analyzed glucose starvation-induced turnover of Hxt1 showed that the two N-terminally located lysine residues at position 12 and 39 are required for ubiquitination and thus degradation [20]. We mutated all N-terminally lysine residues (at positions 12, 27, 35 and 59) and obtained similar results with respect to membrane retention. The mutations improved Hxt1 dependent d-xylose fermentation but overall the effect was smaller than compared to Hxt36. This latter might be due to the poorer *K*_m_ value of Hxt1 for d-xylose, i.e., 880 ± 8 mM [8] *vs* 108 ± 12 mM for Hxt36 [10]. Thus, with Hxt1, the *K*_m_ value is far above the residual d-xylose concentrations at the end of the fermentation (<50 mM), likely causing slow uptake of the d-xylose at the final stages of fermentation leading to incomplete fermentation. The hexose transporter Hxt5 shows a moderate affinity for glucose affinity (10 ± 1 mM [30]), and this transporter is differently regulated as compared to Hxt1 and Hxt3 [36] which are low-affinity glucose transporters and expressed early during fermentations at high glucose concentrations [20, 35]. Hxt5 is mainly expressed at non-fermentable carbon sources and at low growth rates [14, 30]. Also degradation of Hxt5 appears to be different compared to Hxt1 and Hxt36 since in the stationary-phase, after addition of d-glucose, Hxt5 is transiently phosphorylated on serine residues while no ubiquitination could be detected [31]. However, it was proposed that the ubiquitination might have been below the detection limit and therefore ubiquitination could not be excluded. In this respect, the Hxt5 mutant with multiple lysine mutation at the N-terminus as reported in this study, clearly showed improved membrane localization and significantly less vacuolar degradation in the late stages of growth suggesting that ubiquitination may also be involved in the degradation of Hxt5. Nevertheless, this phenomenon has little impact on the growth on solely d-xylose or in anaerobic fermentation with d-glucose and d-xylose. Most likely the amount of Hxt5 on the plasma membrane, in the absence of d-glucose, is still sufficient to maintain the d-xylose uptake and therefore metabolism.

## Conclusions

Membrane localization of the low affinity hexose transporters Hxt1, Hxt36, and Hxt5 is improved by arginine replacement of the N-terminally located lysine residues that are potentially involved in ubiquitination. Interference with ubiquitination results in reduced catabolite degradation and retention of the hexose transporters also in the absence of d-glucose in the medium. Consequently, an improved growth on d-xylose occurs with cells bearing Hxt1 and Hxt36 as sole transporters. The improved growth rate on d-xylose, in the absence of d-glucose, also improves the fermentation time in an industrial-like setting when cells are grown on both d-glucose and d-xylose.

## Methods

### Molecular biology techniques and chemicals

DNA polymerase, restriction enzymes, and T4 DNA ligase were acquired from ThermoFisher Scientific and used following manufacturer’s instructions. Oligonucleotides used for strain constructions were purchased from Sigma-Aldrich (Zwijndrecht, the Netherlands).

### Strains and growth conditions

All *S. cerevisiae* strains used in this study were provided by DSM Bio-based Products & Services and described, in detail, elsewhere [10]. They are made available for academic research under a strict Material Transfer Agreement with DSM (contact: paul.waal-de@dsm.com). In short, the xylose-fermenting *S. cerevisiae* strains are capable of converting xylose into xylulose via an introduced xylose isomerase (XI), whereupon xylulose is phosphorylated into xylulose-5P by xylulose kinase (Xks1). Xylulose-5P then enters the pentose phosphate pathway. In addition, the key enzymes of the pentose phosphate pathway (Tal1, Rpe1, Rki1, and Tki1) are overexpressed. Yeast expression plasmid pRS313 is described elsewhere [36] and modified using the promoter and terminator of Hxt7 [10]. Shake flask experiments at 200 rpm were done in minimal medium supplemented with d-maltose, d-xylose, and d-glucose as indicated. For the fermentation experiments, cells were pregrown on 2 % d-glucose for 16 h and then used to inoculate the main fermentation at a starting OD_600_ of 0.2 using either 3 % d-glucose, 3 % d-xylose or 3 % d-glucose and 3 % d-xylose. Cells were grown anaerobically up to 48 h. Cell growth was monitored by optical density (OD) at 600 nm using an UV–visible spectrophotometer (Novaspec PLUS).

### Analytical methods

High performance liquid chromatography (HPLC from Shimadzu, Kyoto, Japan) was performed using an Aminex HPX-87H column at 65 °C (Bio-RAD) and a refractive index detector (Shimadzu, Kyoto, Japan) was used to measure the concentrations of d-glucose, d-xylose, and ethanol. The mobile phase was 0.005 N H_2_SO_4_ at a flow rate of 0.55 ml/min.

### Cloning of *HXT36*, *HXT1,* and *HXT5* and mutants

The pRS313-P7T7 plasmid, containing the Cen/ARS low copy origin and histidine selection marker, expressing Hxt36 and Hxt36 N367A were used in a previous study [10]. The genes *HXT1* and *HXT5* were amplified on genomic DNA of the DS68616 strain [10] using the primers listed in Additional file 1: Table S1 with the Phusion^®^ High-Fidelity PCR Master Mix with HF buffer. The full-length DNA of *HXT1* and *HXT5* was amplified using primers F HXT1 XbaI, R HXT1 Cfr9I and F HXT5 XbaI and HXT5 Cfr9I, respectively, and cloned into pRS313-P7T7. Overlap PCR with the Phusion^®^ High-Fidelity PCR Master Mix, in which the original *HXTs* in the pRS313-P7T7 plasmid were used as template, was used to amplify the hexose transporters and modify the specified lysines into arginines using the primers in Additional file 1: Table S1. The C-terminal GFP fusions with Hxt36, Hxt1, and Hxt5 and their lysine mutants were made by amplification of the corresponding genes with the Phusion^®^ High-Fidelity PCR Master Mix using the corresponding forward primer (with and without lysine mutations) and the reverse primer without stop codon (Additional file 1: Table S1).

### Fluorescence microscopy

Fresh colonies of the transformants expressing the various variants of *HXT36* in DS68625 were inoculated in duplicates in minimal medium with 2 % d-glucose and grown overnight. Cultures were subsequently inoculated in 2 % d-glucose and d-xylose at a starting OD_600_ of 0.1. To determine the cellular localization, after 0, 16, 24, and 40 h, the fluorescence was analyzed using a Nikon Eclipse-Ti microscope equipped with a 100× oil immersion objective, a filter set for GFP, and a Nikon DS-5Mc cooled camera.

### Uptake measurement

To determine the kinetic parameters of transport, cells were grown for 15 h in shake flasks in minimal medium containing 4 % d-maltose and were collected by centrifugation (3000 rpm, 3 min, 20 °C), washed and re-suspended in minimal medium without carbon source. [^14^C] d-xylose or [^14^C] d-glucose stocks were added to the cell suspension, and the reaction was stopped, after 15 s for d-glucose and 60 s for d-xylose, by addition of 4 ml of ice cold 0.1 M lithium chloride and washed once with the same solution. Samples were filtered over 0.45 μm HV membrane filters (Milipore, France) and counted by Liquid Scintillation Counter (Perkin-Elmer, USA). d-xylose and d-glucose concentrations were varied from 0.5 to 200 mM and 0.1 to 250 mM, respectively.

## Additional file

10.1186/s12936-016-1440-1 Additional data.

## Abbreviations

BP: base pair
GFP: green fluorescence protein
HXT: hexose transporter
OD: optical density
PCR: polymerase chain reaction

## References

[1] Carroll A, Somerville C (2009). Cellulosic biofuels. Annu Rev Plant Biol.

[2] Kotter P, Ciriacy M (1993). Xylose fermentation by *Saccharomyces cerevisiae*. Appl Microbiol Biotechnol.

[3] Tantirungkij M, Seki T, Yoshida T (1994). Genetic improvement of *Saccharomyces cerevisiae* for ethanol production from xylose. Ann N Y Acad Sci.

[4] Kuyper M, Toirkens MJ, Diderich JA, Winkler AA, van Dijken JP, Pronk JT (2005). Evolutionary engineering of mixed-sugar utilization by a xylose-fermenting *Saccharomyces cerevisiae* strain. FEMS Yeast Res.

[5] Kuyper M, Winkler AA, van Dijken JP, Pronk JT (2004). Minimal metabolic engineering of *Saccharomyces cerevisiae* for efficient anaerobic xylose fermentation: a proof of principle. FEMS Yeast Res.

[6] Kuyper M, Harhangi HR, Stave AK, Winkler AA, Jetten MS, de Laat WT, den Ridder JJ, Op den Camp HJ, van Dijken JP, Pronk JT (2003). High-level functional expression of a fungal xylose isomerase: the key to efficient ethanolic fermentation of xylose by *Saccharomyces cerevisiae*?. FEMS Yeast Res.

[7] Hamacher T, Becker J, Gardonyi M, Hahn-Hagerdal B, Boles E (2002). Characterization of the xylose-transporting properties of yeast hexose transporters and their influence on xylose utilization. Microbiology.

[8] Saloheimo A, Rauta J, Stasyk OV, Sibirny AA, Penttila M, Ruohonen L (2007). Xylose transport studies with xylose-utilizing *Saccharomyces cerevisiae* strains expressing heterologous and homologous permeases. Appl Microbiol Biotechnol.

[9] Farwick A, Bruder S, Schadeweg V, Oreb M, Boles E (2014). Engineering of yeast hexose transporters to transport d-xylose without inhibition by d-glucose. Proc Natl Acad Sci.

[10] Nijland JG, Shin HY, de Jong RM, de Waal PP, Klaassen P, Driessen AJ (2014). Engineering of an endogenous hexose transporter into a specific d-xylose transporter facilitates glucose-xylose co-consumption in *Saccharomyces cerevisiae*. Biotechnol Biofuels.

[11] Kruckeberg AL (1996). The hexose transporter family of *Saccharomyces cerevisiae*. Arch Microbiol.

[12] Boles E, Hollenberg CP (1997). The molecular genetics of hexose transport in yeasts. FEMS Microbiol Rev.

[13] Reifenberger E, Boles E, Ciriacy M (1997). Kinetic characterization of individual hexose transporters of *Saccharomyces cerevisiae* and their relation to the triggering mechanisms of glucose repression. Eur J Biochem.

[14] Verwaal R, Paalman JWG, Hogenkamp A, Verkleij AJ, Verrips CT, Boonstra J (2002). HXT5 expression is determined by growth rates in *Saccharomyces cerevisiae*. Yeast.

[15] Ozcan S, Johnston M (1995). Three different regulatory mechanisms enable yeast hexose transporter (HXT) genes to be induced by different levels of glucose. Mol Cell Biol.

[16] Ozcan S, Johnston M (1999). Function and regulation of yeast hexose transporters. Microbiol Mol Biol Rev.

[17] Platt A, Reece RJ (1998). The yeast galactose genetic switch is mediated by the formation of a Gal4p-Gal80p-Gal3p complex. EMBO J.

[18] Kim J-H, Brachet V, Moriya H, Johnston M (2006). Integration of transcriptional and posttranslational regulation in a glucose signal transduction pathway in *Saccharomyces cerevisiae*. Eukaryot Cell.

[19] Lakshmanan J, Mosley AL, Ozcan S (2003). Repression of transcription by Rgt1 in the absence of glucose requires Std1 and Mth1. Curr Genet.

[20] Roy A, Kim Y-B, Cho KH, Kim J-H (2014). Glucose starvation-induced turnover of the yeast glucose transporter Hxt1. Biochim Biophys Acta.

[21] Roy A, Shin YJ, Cho KH, Kim J-H (2013). Mth1 regulates the interaction between the Rgt1 repressor and the Ssn6-Tup1 corepressor complex by modulating PKA-dependent phosphorylation of Rgt1. Mol Biol Cell.

[22] Kim J-H, Polish J, Johnston M (2003). Specificity and regulation of DNA binding by the yeast glucose transporter gene repressor Rgt1. Mol Cell Biol.

[23] Flick KM, Spielewoy N, Kalashnikova TI, Guaderrama M, Zhu Q, Chang H-C, Wittenberg C (2003). Grr1-dependent inactivation of Mth1 mediates glucose-induced dissociation of Rgt1 from HXT gene promoters. Mol Biol Cell.

[24] Rolland F, Winderickx J, Thevelein JM (2002). Glucose-sensing and -signalling mechanisms in yeast. FEMS Yeast Res.

[25] Johnston M, Kim J-H (2005). Glucose as a hormone: receptor-mediated glucose sensing in the yeast *Saccharomyces cerevisiae*. Biochem Soc Trans.

[26] Kim J-H, Roy A, Jouandot D, Cho KH (2013). The glucose signaling network in yeast. Biochim Biophys Acta.

[27] Horák J (2013). Regulations of sugar transporters: insights from yeast. Curr Genet.

[28] Snowdon C, van der Merwe G (2012). Regulation of Hxt3 and Hxt7 turnover converges on the Vid30 complex and requires inactivation of the Ras/cAMP/PKA pathway in *Saccharomyces cerevisiae*. PLoS ONE.

[29] Finley D, Ulrich HD, Sommer T, Kaiser P (2012). The ubiquitin-proteasome system of *Saccharomyces cerevisiae*. Genetics.

[30] Diderich JA, Schuurmans JM, Van Gaalen MC, Kruckeberg AL, Van Dam K (2001). Functional analysis of the hexose transporter homologue HXT5 in *Saccharomyces cerevisiae*. Yeast.

[31] van Suylekom D, van Donselaar E, Blanchetot C, Do Ngoc LN, Humbel BM, Boonstra J (2007). Degradation of the hexose transporter Hxt5p in *Saccharomyces cerevisiae*. Biol Cell.

[32] Shin HY, Nijland JG, de Waal PP, de Jong RM, Klaassen P, Driessen AJM (2015). An engineered cryptic Hxt11 sugar transporter facilitates glucose–xylose co-consumption in *Saccharomyces cerevisiae*. Biotechnol Biofuels.

[33] Romaní A, Pereira F, Johansson B, Domingues L (2015). Metabolic engineering of *Saccharomyces cerevisiae* ethanol strains PE-2 and CAT-1 for efficient lignocellulosic fermentation. Bioresour Technol.

[34] Young EM, Tong A, Bui H, Spofford C, Alper HS (2014). Rewiring yeast sugar transporter preference through modifying a conserved protein motif. Proc Natl Acad Sci USA.

[35] Reifenberger E, Freidel K, Ciriacy M (1995). Identification of novel HXT genes in *Saccharomyces cerevisiae* reveals the impact of individual hexose transporters on glycolytic flux. Mol Microbiol.

[36] Sikorski RS, Hieter P (1989). A system of shuttle vectors and yeast host strains designed for efficient manipulation of DNA in *Saccharomyces cerevisiae*. Genetics.

