# Supplementary material for: Improving pentose fermentation by preventing ubiquitination of hexose transporters in Saccharomyces cerevisiae
Source: Biotechnol Biofuels. 2016 Jul 26;9:158. doi: 10.1186/s13068-016-0573-3 (PMC4962381; doi:10.1186/s13068-016-0573-3)
Supplement: Supplementary file 1 — 10.1186/s12936-016-1440-1 Additional data. [file 13068_2016_573_MOESM1_ESM.docx]

**Supplemental data**

**
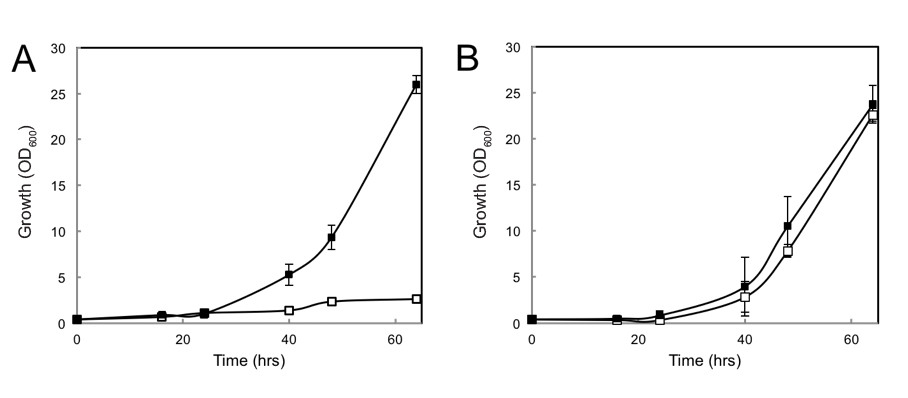
**

**Figure S1.** Growth of the DS68625 strain expressing Hxt1 (A) and Hxt5 (B) on 2 % D-xylose. Depicted as closed squares are the N-terminally lysine mutants (4K and 7K for Hxt1 and Hxt5, respectively) and as open squares for the corresponding parental strains.


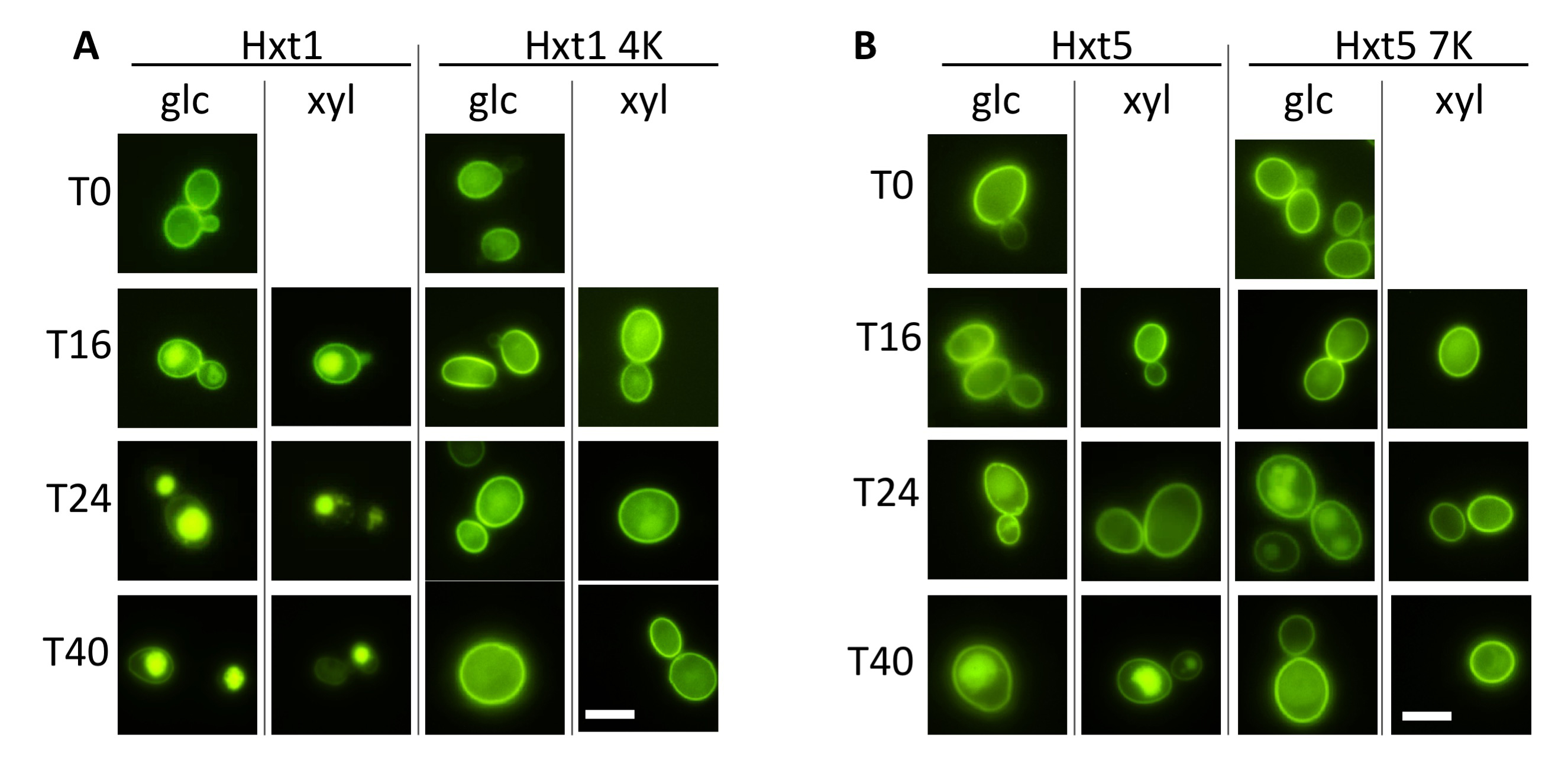


**Figure S2.** Membrane localizationof Hxt1 and Hxt1 4K (A) and Hxt5 and Hxt5 7K (B) fused at the C-terminus to GFP and expressed in strain DS68625 that was grown on minimal medium with 2% D-glucose and 2% D-xylose for a period up to 40 hrs.

**
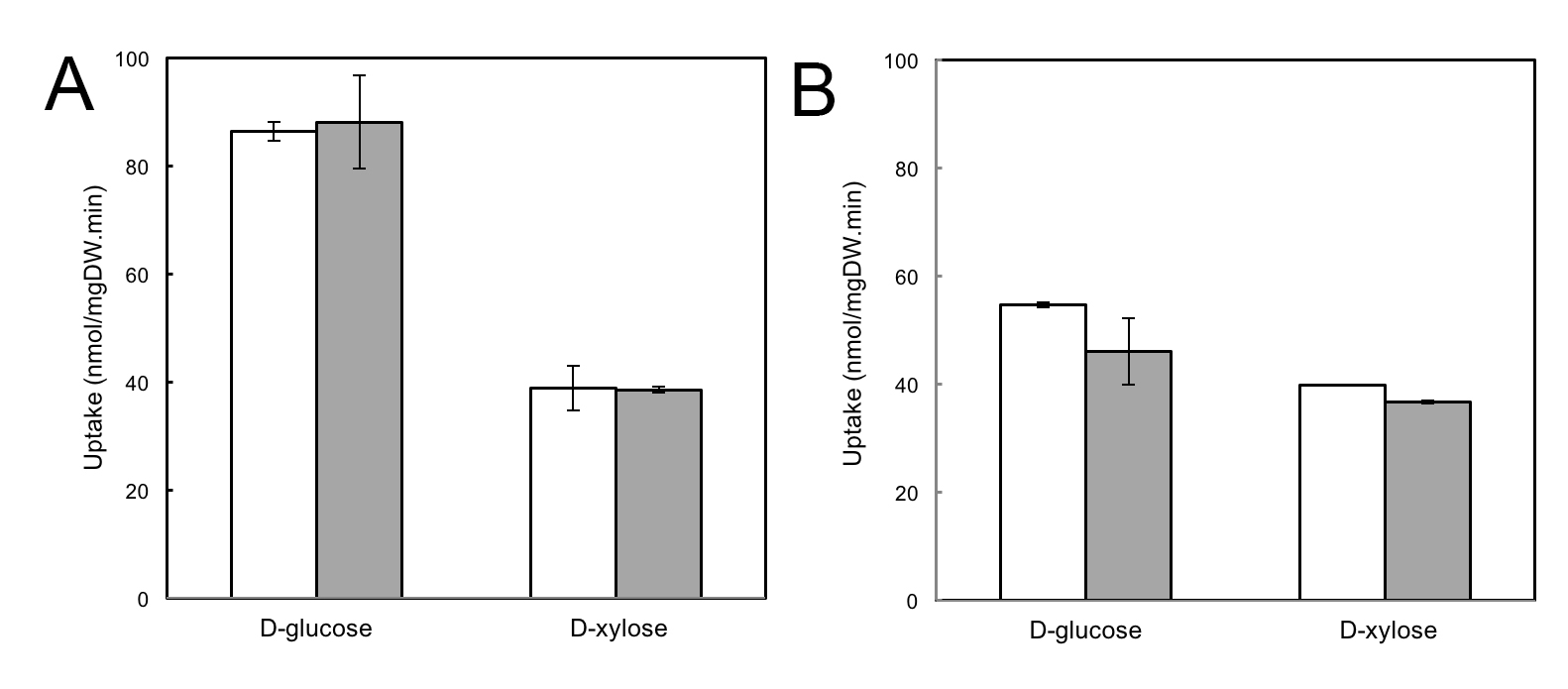
**

**Figure S3.** Uptake of 100 mM D-glucose or D-xylose by strain DS68625 expressing *HXT1* (A) and *HXT5* (B). White bars indicate the wild-type hexose transporters and the grey bars correspond to Hxt1 4K and Hxt5 7K, respectively. Errors are the standard deviation of two independent experiments.


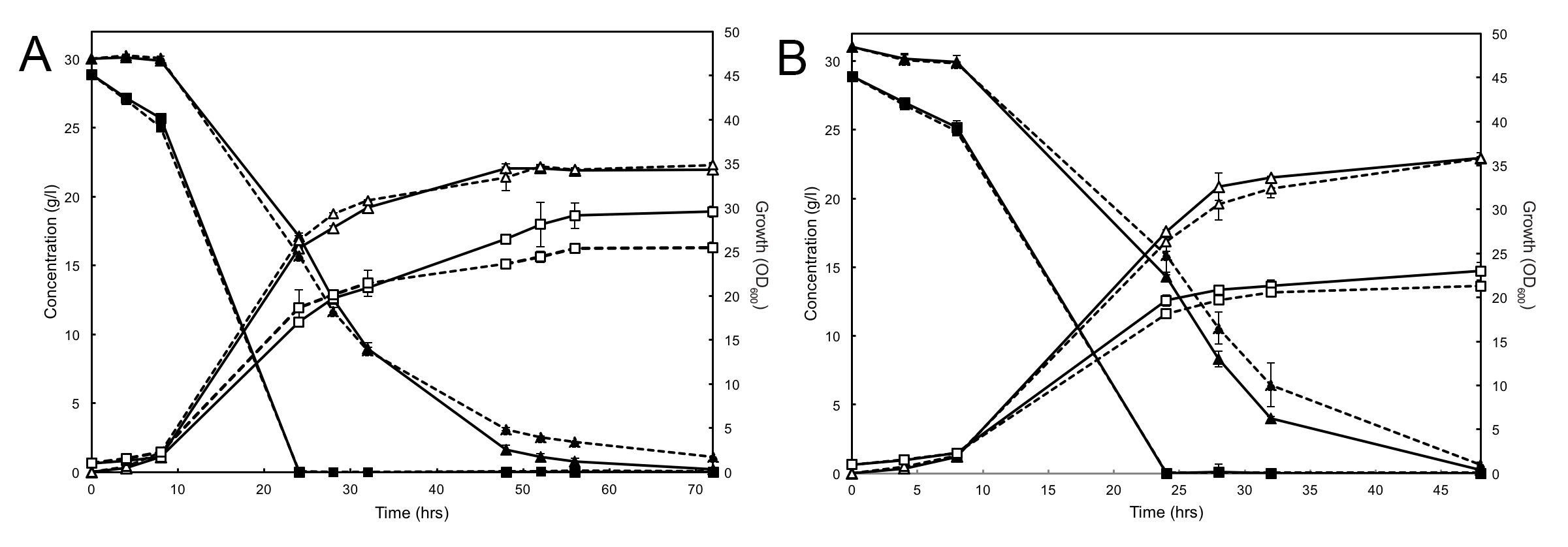


**Figure S4.** Fermentation of D-glucose and D-xylose by strain DS68625 expressing Hxt1 and Hxt1-4K (A) and Hxt5 and Hxt5-7K (B). Symbols depicted show growth (OD_600_; open squares), ethanol (open triangles), D-glucose (closed squares) and D-xylose (closed triangles). The lysine mutants are show as solid lines, and the parental Hxt transporters are indicated with dashed lines. Errors are the standard deviation of two independent experiments.

**Table S1.** Oligonucleotides used for cloning.

| Name | Sequence (5’ 🡪 3’) |
| --- | --- |
| F HXT36 BcuI | GCATACTAGTATGAATTCAACTCCCGATCTAATATC |
| F Hxt36 Bcui A | TCTAGAACTAGTATGAATTCAACTCCAGATTTAATATCTCCACAA**AGG**TCAAGTG |
| R Hxt36 BC | GCACC**TCT**ACCTGTATTTGGGTTGGTAAGTACTTGGTCGGCCTCAGCTTGGAAATCATCTTGAACACC**TCT**TTCTTCAGG |
| F Hxt36 BC | CCTGAAGAA**AGA**GGTGTTCAAGATGATTTCCAAGCTGAGGCCGACCAAGTACTTACCAACCCAAATACAGGT**AGA**GGTGC |
| R Hxt36 DE | GGCAGA**CCT**CCATGGTAGAACACCTTCTTCCCACATGGTGTTGACTTCTTCCAAAGTCAAACC**CCT**AGTTTCTGG |
| F Hxt36 DE | CCAGAAACT**AGG**GGTTTGACTTTGGAAGAAGTCAACACCATGTGGGAAGAAGGTGTTCTACCATGG**AGG**TCTGCC |
| R Hxt36 BamHI | ACGTGGATCCTTATTTGGTGCTGAACATTCTCTTGT |
| R Hxt36 -stop BamHI | CCATGGATCCTTTGGTGCTGAACATTCTCTTGTAC |
| R Hxt36 BamHI FGH | CCGGGGGATCCTTA**TCT**GGTGCTGAACATTCT**CCT**GTACAATGG**CCT**ATCATCGTG |
| R Hxt36-stop BamHI FGH | CCGGGGGATCC**TCT**GGTGCTGAACATTCT**CCT**GTACAATGG**CCT**ATCATCGTG |
| F Hxt1 XbaI | GCATTCTAGAATGAATTCAACTCCCGATCTAATATC |
| F Hxt1 XbaI 1k | AAAATCTAGAATGAATTCAACTCCCGATCTAATATCTCCTCAG**AGA**TCCAATTC |
| R Hxt1 2k3k | CTTTCATT**TCT**ACCTTCTGGAGTATTCATGGC**CCT**TGAACG |
| F Hxt1 2k3k | CGTTCA**AGG**GCCATGAATACTCCAGAAGGT**AGA**AATGAAAG |
| R Hxt1 4k | CGTTACGTAGACACC**TCT**TCCG |
| F Hxt1 4k | CGGA**AGA**GGTGTCTACGTAACG |
| R Hxt1 Cfr9I | GCAGCCCGGGTTATTTCCTGCTAAACAAAC |
| R Hxt1-stop Cfr9I | GCAGCCCGGGTTTCCTGCTAAACAAAC |
| F Hxt5 XbaI | AAAATCTAGAATGTCGGAACTTGAAAACGC |
| R Hxt5 1k | CGAGTTTCCTGA**CCT**CTCGTTG |
| F Hxt5 1k | CAACGAG**AGG**TCAGGAAACTCG |
| R Hxt5 2k3k | CGTC**TCT**GGGAGGGCCTTCATGGGAAATGTAACTTGAGACGGG**TCT**AGC |
| F Hxt5 2k3k | GCT**AGA**CCCGTCTCAAGTTACATTTCCCATGAAGGCCCTCCC**AGA**GACG |
| R Hxt5 4k5k6k7k | CCGA**CCT**CGA**TCTCCT**CTCTAGTTGGTTGTCAACCTC**CCT**CTG |
| F Hxt5 4k5k6k7k | CAG**AGG**GAGGTTGACAACCAACTAGAG**AGGAGA**TCG**AGG**TCGG |
| R Hxt5 Cfr9I | GCAGCCCGGGTTATTTTTCTTTAGTGAAC |
| R Hxt5 -stop Cfr9I | GCAGCCCGGGTTTTTCTTTAGTGAAC |

Underlined, restriction site; **Bold**, introduced mutation.

**Table S2.** Ethanol Yield, production rate and productivity of strain *S. cerevisiae* DS68625 expressing different transporters and grown on mixed and single sugars.

|  | **Hxt36** | **Hxt36-3K** | **Hxt36 N367A** | **Hxt36 N367A-3K** | **Hxt11 *** | **Hxt11 N366T *** |
| --- | --- | --- | --- | --- | --- | --- |
| **3 % D-glucose and 3 % D-xylose** | | | | | | |
| **Y_EtOH_** | 0.39 ± 0.01 | 0.39 ± 0.02 | 0.39 ± 0.01 | 0.39 ± 0.02 | 0.43 ± 0.01 | 0.41 ± 0.02 |
| **Q_EtOH_** | 0.75 ± 0.08 | 0.74 ± 0.06 | 0.74 ± 0.1 | 0.72 ± 0.06 | 0.76 ± 0.02 | 0.72 ± 0.06 |
| **EtOH prod.** | 0.48 ± 0.04 | 0.54 ± 0.03 | 0.53 ± 0.02 | 0.52 ± 0.07 | 1.56 ± 0.35 | 1.36 ± 0.29 |
| **3 % D-xylose** | | | | | | |
| **Y_EtOH_** | 0.39 ± 0.02 | 0.39 ± 0.02 | 0.40 ± 0.02 | 0.40 ± 0.01 | n.d. | n.d. |
| **Q_EtOH_** | 0.09 ± 0.01 | 0.21 ± 0.02 | 0.11 ± 0.04 | 0.23 ± 0.01 | n.d. | n.d. |
| **EtOH prod.** | 0.08 ± 0.03 | 0.27 ± 0.04 | 0.06 ± 0.03 | 0.29 ± 0.05 | n.d. | n.d. |
| **3 % D-glucose** | | | | | | |
| **Y_EtOH_** | 0.38 ± 0.01 | 0.38 ± 0.02 | 0.39 ± 0.02 | 0.39 ± 0.01 | n.d. | n.d. |
| **Q_EtOH_** | 0.55 ± 0.06 | 0.59 ± 0.06 | 0.65 ± 0.02 | 0.60 ± 0.03 | n.d. | n.d. |
| **EtOH prod.** | 0.45 ± 0.02 | 0.42 ± 0.06 | 0.40 ± 0.05 | 0.45 ± 0.02 | n.d. | n.d. |

The ethanol yield (Y_EtOH_ in g/g sugar) was determined for the complete fermentation profile which concerned 48 hours for growth on 3 % D-glucose and 3 % D-xylose, and on 3 % D-xylose alone; and 25 hours on 3 % D-glucose alone. The ethanol production rate (Q_EtOH_ in g/gDW.hr) was determined during the first 25 hours of the fermentations and the maximal ethanol productivity (in g/l.hr) was determined for 3 % D-glucose and 3 % D-xylose, 3 % D-xylose or 3 % D-glucose at time points 41, 41 and 25 hours, respectively.

*Hxt11 and Hxt11 N366T cells were grown on 7 % D-glucose and 3 % D-xylose (32).

n.d., not determined.
